# Comparison Of Four Dietary Scores As Determinants Of Coronary Heart Disease Mortality

**DOI:** 10.1038/s41598-018-33339-5

**Published:** 2018-10-09

**Authors:** Alessandro Menotti, Paolo Emilio Puddu

**Affiliations:** 1 Association for Cardiac Research, Rome, Italy; 2 EA 4650, Signalisation, électrophysiologie et imagerie des lésions d’ischémie reperfusion myocardique, Université de Caen, Normandie, France; 3 Department of Cardiovascular, Respiratory, Nephrological, Anesthesiological and Geriatric Sciences, Sapienza University of Rome, Rome, Italy

## Abstract

We aimed at comparing 2 *a-priori* -Mediterranean Adequacy Index (MAI), the Median Score (MED) - versus 2 *a-posteriori*, -Factor Analysis (FA2) and Principal Components analysis (PC2)- dietary scores in 1214 CHD-free men aged 45–64 belonging to the Italian Rural Areas of the Seven Countries Study examined in 1965 and followed-up for mortality during 40 years. CHD death was the end-point. Collection of dietary history allowed to define 17 main food groups expressed in gr/day and to compare dietary scores, each divided into 3 classes. Kaplan-Meier curves showed higher survival for classes 2 and 3 (healthy) versus class 1 (unhealthy), but the log-rank test was not significant for the 2 *a-priori* scores. Cox proportional hazards models showed similar significant findings comparing class 3 with class 1 in the *a-posteriori* scores FA2 and PC2, with hazards ratios of 0.48 and 0.43, that became 0.65 and 0.53 respectively after adjusting for six specific risk factors for CHD (age, cigarette smoking, systolic blood pressure, serum cholesterol, body mass index and physical activity). Food intake of class 3 in all 4 scores matched rather well the characteristics of the Mediterranean Diet. The poor performance of *a-priori* dietary scores was partly explained by the unexpected and direct relationship of fruit intake with CHD deaths. The superiority of *a-posteriori* dietary patterns could be in turn due to the specificity of the study population. External validations and comparisons of *a-priori* versus *a-posteriori* dietary patterns in larger cohorts, using the same 17 food groups explored here, are urgently needed.

## Introduction

During the last 60 years the relationships of habitual dietary habits with the occurrence of morbid and fatal events have been studied using different procedures. Either single nutrients or food groups were considered in opposition to multiple nutrients or variously combined food groups. Coronary heart disease (CHD) was the morbid condition more frequently explored since it represents the most common cardiovascular disease in the majority of countries, worldwide. Findings were summarized in some review papers dealing with analyses on single individuals^1–3^ or presented as ecological analyses^4–7^.

The most recent studies tended to concentrate on scores derived from the combination of many food groups alone or with selected nutrients.

There are two main approaches in constructing a dietary score: (1) the *a-priori* approach, whereby the score is constructed using the decisions of the investigators in assigning a potentially beneficial or adverse effect to the various components of the score, this on the basis of previous studies, personal opinions, position papers from institutions or groups, etc.; (2) the *a-posteriori* approach, whereby the construction of the score derives from an “objective” mathematical handling of the diet components, mainly based on correlations across food groups and/or nutrients or combining them in regression equations, or estimating unknown variables related to the observed ones; this approach does not assume any aprioristic role of the various components.

Systematic comparisons of the two approaches on the same data-base are rare^8,9^ and this prompted the interest to explore and compare the role of 4 dietary scores, 2 *a-priori* and 2 *a-posteriori*, in a sample of middle aged men followed-up during 40 years for CHD mortality. In fact, these approaches are rather different and a basic question is whether they produce similar or different results when using the same database. The selection of these score types was done based on the information at hand in the datafile we had.

## Material and Methods

### Data collection

The epidemiological material employed here derives from the Italian Rural Areas of the Seven Countries Study of Cardiovascular Diseases (SCS), and refers to the 5-year follow-up field examination held in 1965 on 1564 men aged 45–64 years. The examination included a dietary survey based on a dietary history questionnaire, administered by expert dieticians. Only 1284 questionnaires (82%) were used for this analysis since proved complete and of good quality. Dietary habits were summarized in 17 food groups, that is using the same employed for the construction of the MAI dietary score^5^, as follows: bread, cereals, potatoes, legumes, vegetables, fruit, meat, fish, eggs, milk, cheese, hard fats, vegetable oils (mainly olive oil), sugar, pastry, sugar beverages, alcohol (mainly from wine), all expressed in gr/day.

Four dietary scores were created, replicating the techniques and using the material of the original papers as respectively referenced. The Mediterranean Adequacy Index (MAI score) is an *a-priori* dietary score proposed by Fidanza *et al*.^5^ and based on the diet of a middle-aged male population sample of the rural village of Nicotera (Southern Italy) examined in 1957 and representing the feasibility study of the SCS. This cohort could not be followed-up after that survey, but its diet was similar to that of other Mediterranean population samples of the SCS (such as Montegiorgio in Italy, Dalmatia in Croatia-former Yugoslavia, Crete and Corfu in Greece) that during a follow-up of 25 or more years showed low incidence and mortality from coronary heart diseases (CHD). Therefore, the Nicotera dietary habits were adopted as a guide to construct the MAI.

This score considers 17 food groups, some typical of the Mediterranean diet (bread, cereals, vegetables, legumes, fruit, fish, vegetable oil, wine), and some atypical (meat, milk, cheese, eggs, hard fats, sugar, pastry, sugar-beverages). The typical ones are added up in the numerator of a fraction (ratio) and the atypical in the denominator, all expressed in gr per 4.2 Kilojoules (1000 calories). The MAI is usually transformed into its natural log and this shape used for further analyses.

The Median Score (MED Score) is an *a-priori* dietary score used in a paper of the HALE (Healthy Aging and Life Expectancy) Project of the European Union, that included also part of the Italian cohorts of the SCS^10^ where each food group, adjusted for 4.2 Kilojoules (1000 Kcal) of energy intake, was divided into 2 classes by the median of the distribution. The potentially beneficial ones assumed value 0 if below the median and 1 if above the median, while the potentially dangerous ones assumed value 1 if below the median and 0 if above the median. In this analysis, the potentially beneficial and potentially dangerous food groups were the same used in the numerator and denominator, respectively, of the MAI. Those contributions were added up reaching an overall score ranging 0 to 17, where 0 is worst and 17 is optimal.

The Factor Analysis Score (FA2 score) is an *a-posteriori* dietary score produced using the Exploratory Factor Analysis (EFA) applied to the dietary survey conducted in 1965 on the Italian Rural areas of the SCS, made of 1284 men age 45–64 years, using the same 17 food groups of the MAI, after adjustment for 4.2 Kilojoules (1000 Kcal) of energy intake^11^. Three factors were extracted as suggested by the Kaiser criterion and the Cattel screen plot and the first 2 covered 83% of variance. However, in preliminary Cox models only factor score 2 (derived from factor 2) showed a significant association with the end-point. Therefore, factors 1 and 3 were excluded from further analysis. Factor 2 was defined by variables whose factor loading was 0.30 or more.

The EFA is a statistical reducing technique that estimates one or more underlying unknown factors so that the observed variables are linear combinations of the factors themselves that influence the observed variables. The EFA tends to account for common variance of the data.

The Principal Component score (PC2 score) is an *a-posteriori* dietary score produced using the Principal Component Analysis (PCA) applied to the dietary survey conducted in 1965 on the Italian Rural areas of the SCS, made of 1284 men aged 45–64 years and quoted in the same paper dealing with Factor Analysis^11^. The same 17 food groups used for the MAI were employed in this analysis after adjustment for 4.2 Kilojoules (1000 Kcal) of energy intake.

The procedure and the outcome were practically the same as for Factor Analysis and only Factor 2 (component 2) was retained for further analysis. Again, Factor 2 (component 2) was defined by variables whose factor loading was 0.30 or more.

The PCA is a statistical reducing technique that produces one or more uncorrelated Components representing many original variables, linearly combined after proper weighting, that explain the maximum amount of variance of the data.

For both Factor and Principal Components Analyses, Varimax rotation was adopted. PCA and EFA are rather similar techniques that are frequently confused with each other because the analytical procedures are very similar, as well as the majority of their terms. In principle, they are very different but the outcome is almost the same when the error terms in the factor analysis model (the variability not explained by common factors) can be assumed to have all the same variance.

Six risk factors measured at the same baseline were considered as possible confounders in the Cox models, as follows: a) age in years approximated to the nearest birthday; b) smoking habits expressed as the number of cigarettes smoked per day; for ex- and never smokers the amount of cigarettes was 0 since preliminary tests showed a similar risk for ex- and never smokers in this long follow-up period of 40 years; c) systolic blood pressure expressed in mmHg, measured at the right arm in supine posture, at the end of a medical examination, following the technique reported in the WHO Cardiovascular Survey Methods Manual^12^: the average of 2 consecutive measurements were used for analysis; d) serum cholesterol, expressed in mmol/L, measured on casual blood samples following the technique of Anderson and Keys^13^; e) body mass index (BMI) expressed as kg/m^2^, following the procedures reported in the WHO Cardiovascular Survey Methods Manual^12^; f) physical activity at work derived from a simple questionnaire matched with the reported profession, divided into 3 classes (sedentary, moderate, vigorous): sedentary physical activity was used a reference in the Cox models.

At entry examination, standardized medical questionnaire, physical examination and ECG recording allowed to identify subjects already carriers of a major prevalent CHD following the criteria of the Seven Countries Study of Cardiovascular Diseases^14^.

Follow-up for mortality was complete for the next 40 years and there was no loss to follow-up. Causes of death were allocated by reviewing and combining information from death certificates, hospital and medical records, interviews with physicians and relatives of the deceased and any other witnesses of fatal events and were determined by a single reviewer following defined criteria, employing the 8^th^ Revision of the WHO-ICD (ICD-8)^15^. In the presence of multiple causes of death (in about half of cases) and uncertainties about the principal cause of death, a hierarchical preference rank was adopted with violence, cancer in advanced stages, CHD, stroke and other in that order.

CHD fatalities were represented by cases manifested as myocardial infarction, other acute ischemic attacks, and cases of sudden coronary death occurring within 2 hours from onset of symptoms, after the reasonable exclusion of other possible causes. Cases manifested only as heart failure, chronic arrhythmia or blocks or with simple mention of chronic CHD, in the absence of typical coronary syndromes, were not included in this group for reason given elsewhere^16,17^.

The field examination was held only a few months after the Helsinki Declaration and acceptance from the subjects was implied in participation. All methods were carried out in accordance with relevant guidelines and regulations at the time of the study start, although institutional or licensing committees were not still present in the Country and accordingly were not consulted. Informed consent was obtained from all subjects during subsequent examinations.

### Statistical analysis

Analysis was run after excluding 70 subjects who had a major prevalent CHD condition, thus reducing the cohort from 1284 to 1214 and the number of CHD fatalities from 225 to 200.

Each dietary score was divided into 3 classes whose levels were low (class 1), intermediate (class 2) or high (class 3). Tertile classes were adopted for MAI, FA2 and PC2 because these scores are continuous variables (with 404, 405 and 405 men in the 3 tertiles, respectively). In the case of MED, whose levels are discrete, arbitrary classes were created with 337 subjects (28%) in class 1 (score levels 0 to 7), 372 (31%) in class 2 (score levels 8 and 9), and 505 (41%) in class 3 (score levels of 10 and above).

Kaplan-Meier survival curves, with the relative log-rank tests, were created for each dietary score divided into 3 classes, with CHD mortality in 40 years as end-point.

Cox proportional hazards models were computed with CHD deaths as dependent variable, classes 2 and 3 of each score as covariates (class 1 as reference) and months of follow-up as time scale. Models were first produced with dietary scores alone, and then by adding age, cigarettes smoking, systolic blood pressure and serum cholesterol and, subsequently, also BMI and physical activity. In each score, multivariable coefficients of class 3 were compared to those of class 1 using the T test.

Food groups intake in 3 classes of each dietary score were computed and tabulated and T test used to compare class 3 with class 1. Significance was judged based on p < 0.05.

Factor loadings for each food group were inserted in the Tables dealing with *a-posteriori* dietary score.

## Results

The 4 dietary scores had largely different means, due to different computational procedures and the absence of real units of measurements. However, variance of the 2 *a-posteriori* scores (FA2 and PC2) was much larger than that of the 2 *a-priori* scores (MAI and MED).

**Figure 1:**
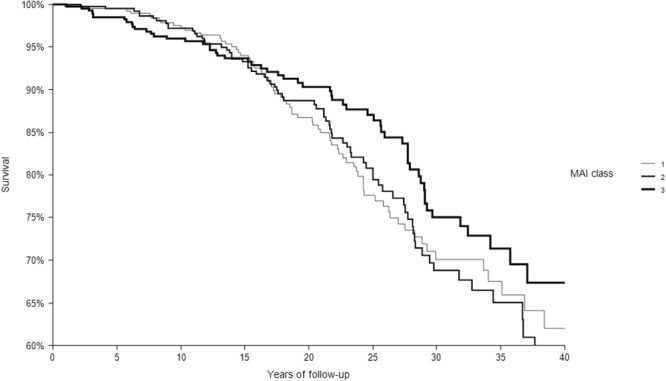
Kaplan-Meier survival curves of CHD mortality in 40 years as a function of 3 classes of MAI dietary score. P of chi-squared log-rank test = 0.3042. Class 1 = low levels; Class 2 = intermediate levels; Class 3 = high levels.

**Figure 2:**
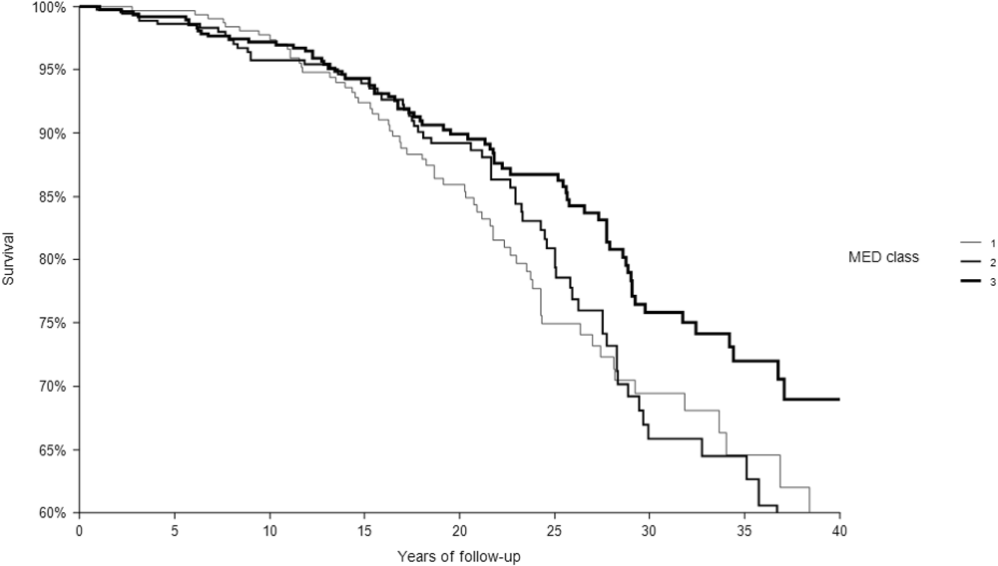
Kaplan-Meier survival curves of CHD mortality in 40 years as a function of 3 classes of MED dietary score. P of chi-squared log-rank test = 0.0651. Class 1 = low levels; Class 2 = intermediate levels; Class 3 = high levels.

**Figure 3:**
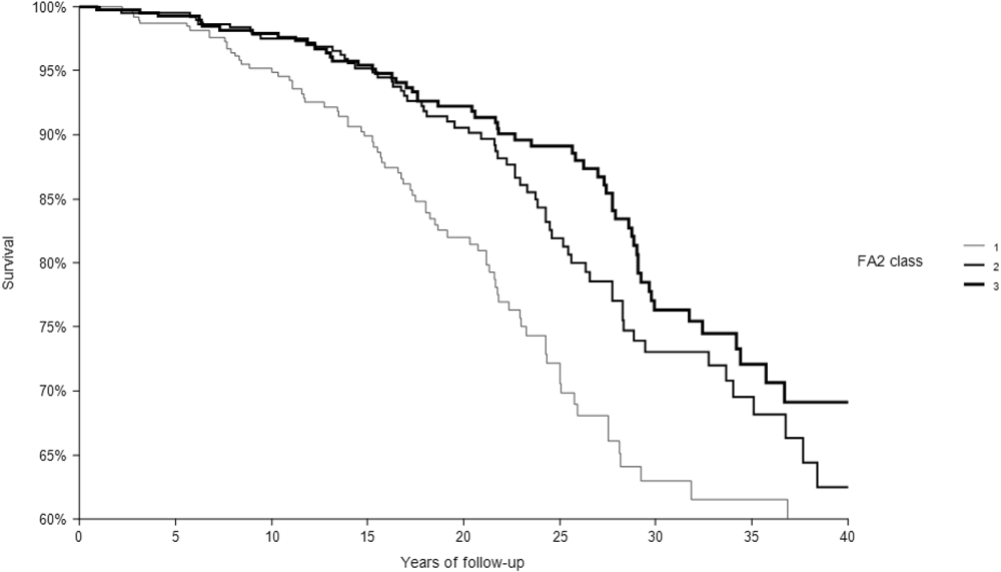
Kaplan-Meier survival curves of CHD mortality in 40 years as a function of 3 classes of FA2 dietary score. P of chi-squared log-rank test <0.0001. Class 1 = low levels; Class 2 = intermediate levels; Class 3 = high levels.

**Figure 4:**
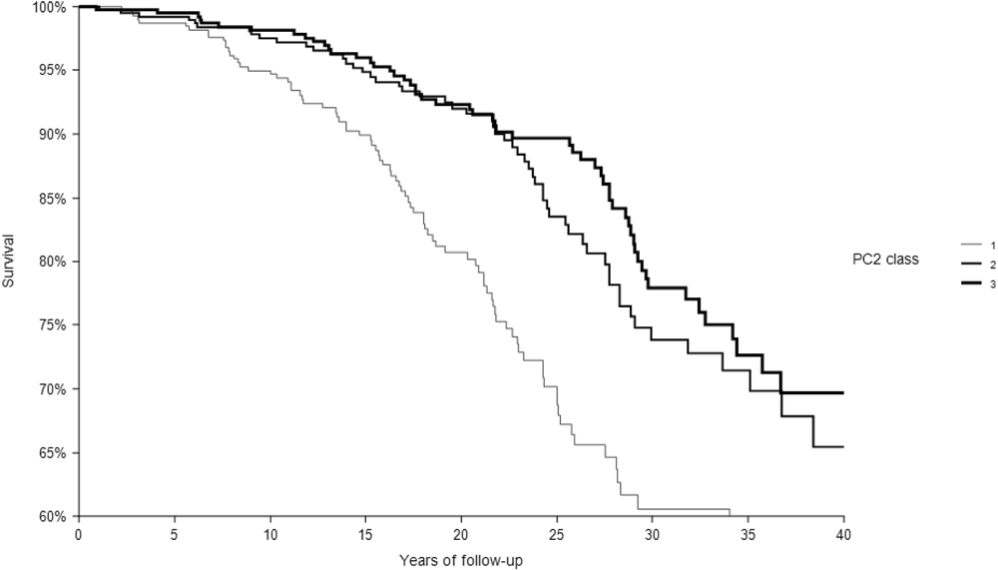
Kaplan-Meier survival curves of CHD mortality in 40 years as a function of 3 classes of PC2 dietary score. P of chi-squared log-rank test <0.0001. Class 1 = low levels; Class 2 = intermediate levels; Class 3 = high levels.

In 40 years of follow-up, among the 1214 CHD-free men enrolled at baseline 1194 died from all-causes, 200 from CHD. The relationships of the 3 classes of each dietary score with CHD deaths are depicted in Figs 1–4 that report Kaplan-Meier survival curves. In all cases, subjects in class 3 had the longest survival, those in class 1 the shortest, while an intermediate survival applied to men in class 2. However, the segregation among the 3 curves was much clearer for FA2 and PC2 *a-posteriori* scores than for the MAI and MED *a-priori* scores. This was confirmed by the p values of the chi-square for the log-rank test that was not significant for the MAI (p = 0.3042), close to significance for MED (p = 0.0651), and highly significant for FA2 and PC2 (both, p < 0.0001).

In a series of Cox proportional hazards models, classes 2 and 3 of each dietary score were used as covariates (class 1 used as reference) to predict CHD mortality in 40 years (Table 1, Panel A). In all cases hazards ratios showed a favorable outcome for class 3 and, to a lesser extent, for class 2, compared to class 1. Hazards ratio (HR) for class 3 ranged 0.80 for MAI to 0.43 for PC2. However, 95% confidence intervals exceeded 1 for both MAI and MED.

**Table 1:** Forty-year CHD mortality as a function of 4 dietary scores in Cox proportional hazards models.

Dietary Scores and Variables in Model	Hazards Ratios (95% Confidence Limits)		
	Risk Class 1	Risk Class 2	Risk Class 3
**Panel A–Original Dietary Scores**			
**MAI**			
Dietary score alone	1	1.03 (0.74 1.42)	0.80 (0.56 1.13**)**
Adjusted for age, cigarettes, systolic blood pressure, serum cholesterol	1	1.03 (0.74 1.43)	0.80 (0.56 1.14)
Adjusted for age, cigarettes, systolic blood pressure, serum cholesterol, BMI, physical activity	1	1.07 (0.77 1.49)	0.87 (0.60 1.25)
**MED**			
Dietary score alone	1	0.97 (0.69 1.38)	0.70 (0.50 0.98)
Adjusted for age, cigarettes, systolic blood pressure, serum cholesterol	1	0.99 (0.70 1.41)	0.74 (0.53 1.05)
Adjusted for age, cigarettes, systolic blood pressure, serum cholesterol, BMI, physical activity	1	1.02 (0.72 1.45)	0.78 (0.55 1.11)
**FA2**			
Dietary score alone	1	0.60 (0.43 0.83)	0.48 (0.34 0.68
Adjusted for age, cigarettes, systolic blood pressure, serum cholesterol	1	0.72 (0.51 1.01)	0.63 (0.45 0.90)
Adjusted for age, cigarettes, systolic blood pressure, serum cholesterol, BMI, physical activity	1	0.72 (0.51 1.00)	0.65 (0.45 0.94)
**PC2**			
Dietary score alone	1	0.51 (0.37 0.72)	0.43 (0.31 0.60)
Adjusted for age, cigarettes, systolic blood pressure, serum cholesterol	1	0.60 (0.43 0.84)	0.54 (0.38 0.76)
Adjusted for age, cigarettes, systolic blood pressure, serum cholesterol, BMI, physical activity	1	0.60 (0.43 0.85)	0.53 (0.37 0.77)
**Panel B - Modified Dietary Scores**			
**MAI, after exclusion of alcohol, sugar beverages and fruit**			
Dietary score alone	1	1.00 (0.72 1.30)	0.64 (0.49 0.98)
Adjusted for age, cigarettes, systolic blood pressure, serum cholesterol	1	1.02 (0.74 1.42)	0.78 (0.55 1.11)
Adjusted for age, cigarettes, systolic blood pressure, serum cholesterol, BMI, physical activity	1	1.07 (0.77 1.49)	0.84 (0.58 1.20)
**MED, after exclusion of alcohol, sugar beverages and fruit**			
Dietary score alone	1	0.96 (0.67 1.36)	0.70 (0.50 0.97)
Adjusted for age, cigarettes, systolic blood pressure, serum cholesterol	1	1.04 (0.79 1.49)	0.80 (0.56 1.13)
Adjusted for age, cigarettes, systolic blood pressure, serum cholesterol, BMI, physical activity	1	1.08 (0.75 1.55)	0.85 (0.59 1.21)

When potentially confounding variables were added, the magnitude of hazards ratios for class 3 became smaller for all scores, but they were still significant for FA2 and PC2 whose final HRs were 0.65 and 0.53 respectively. This confirmed the different predictive power of the 4 scores already suggested by the Kaplan-Meier survival curves. One of the reasons for the loss of predictive power when confounding variables were included into the models was likely due to the correlations of some risk factors with the score themselves. In particular, the correlation coefficients of systolic blood pressure and serum cholesterol with the 2 a*-posteriori* scores were around 0.20 and 0.15, respectively, while they were negligible for the 2 *a-priori scores*, mainly for the MAI. However, a relatively high correlation coefficient was found between MED score and cigarette smoking (R = 0.15).

We found high correlation coefficients between FA2 and PC2 (R = 0.99) scores, smaller correlation between MED and MAI (R = 0.68) scores, even smaller between the *a-posteriori* versus the *a-priori* scores (all R < 0.30).

Distribution of food groups intake in the 3 classes of the 4 dietary scores are reported in detail in Tables 2–5. Moreover, in Tables 4 and 5 factor loadings of Factor Analysis and Principal Components analysis are also reported.

**Table 2:** Food groups (adjusted by energy) as identified by the dietary score MAI divided into 3 tertile classes.

	Class 1 average	Class 2 average	Class 3 average	Ratio Class 3/Class 1	P of t between Class 1 and Class 3
bread	101.2	110.7	128.5	1.27	<0.0001
cereals	39.4	41.5	39.6	1.01	0.8473
potatoes	7.1	8.7	8.4	1.18	0.0254
vegetables	15.5	19.4	21.3	1.37	<0.0001
legumes	1.8	1.2	1.2	0.68	0.1133
fruit	81.4	63.8	42.5	0.52	<0.0001
oils	13.0	14.0	13.1	1.01	0.8202
fish	6.4	7.2	8.2	1.29	<0.0001
alcohol	192.8	273.5	351.0	1.82	<0.0001
meat	51.3	43.2	21.9	0.43	<0.0001
eggs	5.9	6.3	7.0	0.88	0.6636
fat	7.4	7.1	7.5	1.01	0.7943
milk	90.2	14.1	0.6	0.01	<0.0001
cheese	6.2	4.9	2.6	0.41	<0.0001
sugar	7.9	4.2	1.4	0.18	<0.0001
pastry	7.5	3.7	0.9	0.12	<0.0001
alcohol	192.8	273.5	351.0	1.82	<0.0001
sugar beverages	0.4	0.2	0.1	0.20	<0.0001

**Table 3:** Food groups (adjusted by energy) as identified by the dietary score MED divided into 3 arbitrary classes.

	Class 1 average	Class 2 average	Class 3 average	Ratio Class 3/Class 1	P of t between Class 1 and Class 3
bread	99.6	111.8	123.9	1.24	<0.0001
cereals	37.3	39.9	42.3	1.13	<0.0001
potatoes	5.8	7.4	10.0	1.71	<0.0001
vegetables	12.6	17.1	24.1	1.92	<0.0001
legumes	1.4	1.0	1.7	1.17	0.4264
fruit	78.3	69.2	47.1	0.60	<0.0001
oils	11.9	13.9	14.1	1.19	<0.0001
fish	5.1	7.0	9.2	1.83	<0.0001
alcohol	240.5	267.5	297.5	1.24	<0.0001
meat	50.3	41.1	29.4	0.58	<0.0001
eggs	7.0	5.8	5.0	0.72	<0.0001
fat	8.2	7.0	7.0	0.85	0.0015
milk	69.0	37.9	10.0	0.14	<0.0001
cheese	6.2	5.1	3.1	0.50	<0.0001
sugar	7.6	4.8	2.2	0.29	<0.0001
pastry	8.1	3.9	1.4	0.17	<0.0001
sugar beverages	0.5	0.2	0.1	0.09	<0.0001

**Table 4:** Food groups (adjusted by energy) as identified by factor analysys FA2 dietary score divided into 3 tertile classes.

	Class 1 average	Class 2 average	Class 3 average	Ratio Class 3/Class 1	P of t between class 1 and class 3	Factor loading
bread	99.0	115.7	125.6	1.27	<0.0001	0.4787*
cereals	35.6	40.7	44.2	1.24	<0.0001	0.4538*
potatoes	5.5	7.1	11.5	2.10	<0.0001	0.3481*
vegetables	12.7	18.3	25.3	1.99	<0.0001	0.4139*
legumes	1.8	1.3	1.1	0.61	0.0587	0.0405
fruit	91.7	56.3	39.7	0.43	<0.0001	−0.1369
oils	13.2	14.3	12.6	0.96	0.1621	0.2328
fish	5.1	7.1	9.6	1.88	<0.0001	0.3658*
alcohol	342.2	247.2	228.3	0.67	<0.0001	0.0706
meat	43.8	39.8	32.8	0.75	<0.0001	0.1294
eggs	6.1	5.5	5.7	0.93	0.4136	0.1565
fat	6.8	7.0	8.2	1.20	0.0003	0.2482
milk	53.3	34.5	17.0	0.32	<0.0001	−0.1581
cheese	4.1	4.8	4.8	1.16	0.3407	0.1323
sugar	5.9	4.6	3.0	0.51	<0.0001	−0.1144
pastry	3.8	4.8	3.5	0.90	0.5702	0.0176
sugar beverages	0.2	0.3	0.2	0.96	1.000	−0.2260

*Food groups defining Factor 2.

**Table 5:** Food groups (adjusted by energy) as identified by Principal Component analysis, component PC2 dietary score divided into 3 tertile classes.

	Class 1 average	Class 2 average	Class 3 average	Ratio Class 3/Class 1	P of t between Class 1 and Class 3	Factor loading
bread	100.9	115.2	124.3	1.23	<0.0001	0.5826*
cereals	37.2	39.7	43.5	1.17	<0.0001	0.5952*
potatoes	5.5	7.1	11.5	2.11	<0.0001	0.4498*
vegetables	13.9	17.3	25.0	1.79	<0.0001	0.5503*
legumes	1.8	1.2	1.2	0.64	0.1051	0.0215
fruit	93.0	55.7	38.9	0.42	<0.0001	−0.1816
oils	13.4	14.1	12.7	0.95	0.2220	0.3468*
fish	5.2	6.8	9.9	1.89	<0.0001	0.5021*
alcohol	312.6	260.3	244.5	0.78	<0.0001	0.0719
meat	44.4	39.7	32.4	0.73	<0.0001	0.1702
eggs	5.7	5.6	6.0	1.06	0.5366	0.1716
fat	6.6	7.1	8.3	1.25	<0.0001	0.3550*
milk	54.2	35.2	15.3	0.28	<0.0001	−0.1696
cheese	4.4	4.5	4.8	1.08	0.3601	0.2191
sugar	6.1	4.6	2.8	0.45	<0.0001	−0.1149
pastry	4.3	4.8	3.0	0.70	<0.0001	0.0480
sugar beverages	0.2	0.3	0.1	0.50	0.0634	0.0080

*Food groups defining Factor 2.

In the upper part of Tables 2–5 there are food groups declared “beneficial” by the *a-prori* dietary scores, while asterisks in Tables 4 and 5 label food groups that contributed to the identification of factor score 2 in Factor Analysis and Principal Component Analysis, respectively. They were bread, cereals, potatoes, vegetables and fish in Factor Analysis and the same plus oils and fat in Principal Components Analysis. All of them, except fat, were labelled as “beneficial “ in the *a-priori* scores, and therefore there were some common features to all scores, since significantly higher intakes of bread, potatoes, vegetables and fish were found in class 3 than in class 1, while the reverse was true for meat, milk and sugar that were more consumed in class1 than in class 3 (on average an excess of 61% for meat, 533% for milk and 280% for sugar).

Another common feature was the direct unexpected relationship of fruit consumption with CHD mortality risk. For some other food groups there were differences across the 4 dietary scores. In particular, fat intake was higher while alcohol intake was lower in class 3 compared with class 1 of the *a-posteriori* scores, while the reverse was the case, for alcohol intake, in the *a-priori* scores. In particular, the choice of the “beneficial” food groups of the *a-priori* scores was not fully confirmed since no significant difference was found for legumes while the role of fruit was contrary to what expected.

On the other hand, the 5 and 7 food groups chosen for the identification of factor 2 in the two *a-posteriori* scores (FA2 and PC2, respectively) were among those selected as “beneficial “ by the MAI and the MED scores. In general, class 3 of all scores roughly depicted the structure of the so-called Mediterranean Diet, rich in vegetable food, vegetable oils and fish (but not fruit) while in class 1 consumption of those food groups was lower.

The limited predictive power of the 2 *a-priori* scores, MAI and MED, could have been bound to the presence of fruit among the food groups classified as “beneficial” while in this material and analysis it was directly related to CHD risk. This could be attributed to the high direct correlation (R > 0.20) of fruit intake with intake of meat, milk and sugar and the inverse correlation with bread intake. Therefore, in a sensitivity analysis we concomitantly excluded fruit (because performed in an opposite way compared with the expectation), alcohol (because of the excessive quantities compared with all other food groups) and sugar beverages (because used by a minimal number of subjects). The outcome is reported in Table 1, Panel B where HRs of class 3 of MAI and MED became statistically significant and maintained the right direction while losing significance when 6 other covariates were added.

The whole analysis was replicated using the full sample of 1284 men, including those with prevalent CHD, and findings were similar although slightly less relevant. We found that the dietary score of the CHD prevalent subjects (for example using FA2 score) was significantly lower (p < 0.0001) than for subjects free from CHD suggesting that, despite the occurrence of a previous non-fatal event, they maintained a less healthy diet than that of the CHD-free men. Of note, death rates from CHD in CHD prevalent men was about twice than among the CHD-free men (36% versus 17%).

In a separate test, we used 40-year all-cause mortality as end-point reaching similar results, that is a better performance of the *a-posteriori* scores compared with the *a-priori* scores (data not shown).

## Discussion

The 4 dietary scores compared here were associated in the same direction with CHD long-term mortality but with different strength. The 2 *a-posteriori* scores performed better than the 2 created *a-priori*, for reasons that cannot be easily envisaged.

The common characteristic to all scores was that they were inversely related with CHD risk, and therefore class 3 (the highest) was protective against CHD deaths, but with different strengths across the 4 scores. Class 3 of all scores lost significance when the Cox models included some selected covariates strongly related to CHD and at the end only class 3 of FA2 and PC2 scores remained significantly associated with the outcome.

The same high levels (those in class 3) of the 4 scores roughly described the characteristics of the Mediterranean Diet, with higher intake of vegetable foods, oils and fish than the other classes and class 1 in particular.

In score class 3 (the healthy one) of FA2 and PC2, there was a significant excess consumption of hard fat compared to class 1. However, this was largely counterbalanced by the high consumption of vegetable oils that was a general characteristic of this population. In fact, 82% of men had a ratio of vegetable oils to hard fat largely greater than 1 (in a few cases equal to 1), suggesting that, overall, this is a Mediterranean Diet consuming population. Based on a rough computation of vegetable oils (in the majority of cases olive oil) and hard fat (mainly butter but with a sizable proportion of lard) the ratio of unsaturated fat to saturated fat was about 5.

The performance of the MAI was different from a previous analysis carried out in the same material, but in that case the definition of the end-point was somewhat different and subjects with exceedingly high level of MAI bound to enormous wine consumption were excluded. The average wine consumption was very high in the whole population, with a median of 750 ml/day^18^.

It is difficult to explain why the *a-posteriori* scores performed better than the *a-priori* scores in their association with CHD mortality. Moreover, we had to cope with the apparently adverse effect of fruit consumption whose role was unexpected and contrary to what commonly found. In fact, an excess of fruit consumption was always found in Class 1 versus Class 3 and the consequent excess of fructose might have risen blood levels of serum cholesterol and LDL lipoproteins as suggested in some studies^19^. After having explored the problem in more detail, the following facts became clear: i) men eating more fruit were at higher CHD risk when fruit alone was fed as a covariate in a Cox model (Hazard ratio = 1.0583; 95% confidence limits = 1.0028 and 1.1168); ii) class 3 of all scores (still being at a lesser CHD risk) was the one with the lowest fruit intake; iii) high direct correlations were found between fruit intake and meat, milk and sugar intake (R > 0.20), that is a higher intake of “unhealthy” food groups among those eating more fruit, and an inverse correlation was found with bread intake, facts that provide a partial explanation of these “abnormal” findings due to possible confounding with other unhealthy foods; iv) finally, all this could be a “specificity” of this population.

However, this is not a unique case since in a SCS report dealing with ecological analysis of 16 cohorts and a 25-year follow-up, CHD risk was not associated with fruit intake^4^.

Another choice that could have hampered the analysis was to put wine (and alcohol) in the list of “beneficial” food groups of the MAI, because in this population the wine consumption was extremely high as mentioned above. The sensitivity analysis partially answered these suspects. In fact, in the *a-posteriori* analysis alcohol intake was lower in class 3 than in class 1, while the opposite occurred in the *a-priori* analysis.

Although the *a-posteriori* scores seem theoretically superior in their association with CHD mortality, the *a-priori* scores have the advantage to be practically applied to other populations in an easy way once the same food groups intake is known. This is not the case for the *a-posteriori* scores since they represent only the structure of the study population. In fact, it is not clear, or at least not demonstrated, which procedure should be used to convert the findings of FA2 and PC2 into a practical predictive system. Attempts could be made using the Factor coefficients and the Component coefficients but, again, this exercise needs the availability of another population, with the same food groups and the same end-point as a minimum.

Other well-established dietary scores could have been tested, such as the MDS from Trichopoulou *et al*.^20^, but some of its components were not available in our database (in particular nutrients represented by the various types of fat).

Reports on *a-priori* dietary scores in relation to CHD or cardiovascular diseases are relatively frequent in the literature. A recent review identified 26 studies dealing with 5 original indexes, plus 10 indexes modified from other scores^2^. In general, most of them provided evidence that diet patterns defined “Healthy”, or “Prudent” or “Mediterranean” were associated with lower incidence or mortality from CHD, and frequently other causes of death or all-cause mortality.

Reports on *a-posteriori* scores are less common^21–26^ and usually they employed Factor Analysis, less frequently Principal Component Analysis. Those quoted here dealt with different end-points such as hypertension, CHD and stroke and were related to dietary patterns defined Prudent Diet or Mediterranean Diet. CHD incidence or mortality showed an inverse relationship with patterns representing the Mediterranean Diet, less frequently a direct relationship with a Western type Diet.

A review of 12 studies on *a-posteriori* dietary scores versus CHD conducted in the US, Europe and Asia, showed some inconsistencies^3^. After careful evaluation of data and procedures, the conclusion was that non-standardized methods, different sample characteristics, follow-up periods and definition of events could largely explain the discrepancies. In any case, diet scores derived from *a-posteriori* procedures and defined as Prudent Diet were protective from CHD incidence.

Two reports dealt, instead, with the comparison of an *a-priori* with an *a-posteriori* dietary score and both of them used the Principal Component Analysis for the *a-posteriori* score and the Med-Diet-Score for the *a-priori* analysis. One of the 2 was based on a population study with 5-year incidence of cardiovascular diseases^8^ while the other one was a case-control study of CHD and stroke prevalence^9^. In both cases, the performance of the 2 patterns was rather similar when tested with the ROC curves and C statistics. This is in contrast with our findings whereby the *a-posteriori* patterns performed better than the *a-priori* patterns. Of note, however, that fruit is considered beneficial in the Trichopoulou *et al*. Med Diet Score although alcohol is only considered beneficial within a limited range^20^. A further explanation could be related to the small size of our study population but also to the high correlation of fruit intake with intake of other unhealthy food groups.

In general, although the superiority of the *a-posteriori* score is not granted based on these findings, their performance is still impressive with HRs that matches those of studies based on much larger samples. Moreover, the construction of the *a-posteriori* scores is independent from the knowledge of the possible role of the single food groups in their relationship to morbid or fatal events, being simply derived from the association among the various food groups analyzed with different approaches.

In conclusion, it might be of interest to produce further comparisons of *a-priori* versus *a-posteriori* dietary patterns studied in the same populations and using the same food groups, standing the uncertainties about their comparability. Moreover, another important external validation may be represented by the application of factor or components coefficients of the *a-posteriori* scores to populations other than those producing the scores.
